# Fucosylation Is a Promising Target for Cancer Diagnosis and Therapy

**DOI:** 10.3390/biom2010034

**Published:** 2012-01-30

**Authors:** Eiji Miyoshi, Kenta Moriwaki, Naoko Terao, Cheng-Cheng Tan, Mika Terao, Tsutomu Nakagawa, Hitoshi Matsumoto, Shinichiro Shinzaki, Yoshihiro Kamada

**Affiliations:** Osaka University Graduate School of Medicine, Department of Molecular Biochemistry and Clinical Investigation, 1-7 Yamada-oka, Suita 565-0871, Japan

**Keywords:** fucosylation, haptoglobin, TRAIL, cancer, oligosaccharide

## Abstract

Oligosaccharides, sequences of carbohydrates conjugated to proteins and lipids, are arguably the most abundant and structurally diverse class of molecules. Fucosylation is one of the most important oligosaccharide modifications involved in cancer and inflammation. Recent advances in glycomics have identified several types of glyco-biomarkers containing fucosylation that are linked to certain types of cancer. Fucosylated alpha-fetoprotein (AFP) is widely used in the diagnosis of hepatocellular carcinoma because it is more specific than alpha-fetoprotein. High levels of fucosylated haptoglobin have also been found in sera of patients with various carcinomas. We have recently established a simple lectin-antibody ELISA to measure fucosylated haptoglobin and to investigate its clinical use. Cellular fucosylation is dependent upon fucosyltransferase activity and the level of its donor substrate, guanosine diphosphate (GDP)-fucose. GDP-mannose-4,6-dehydratase (GMDS) is a key enzyme involved in the synthesis of GDP-fucose. Mutations of GMDS found in colon cancer cells induced a malignant phenotype, leading to rapid growth in athymic mice resistant to natural killer cells. This review describes the role of fucosylated haptoglobin as a cancer biomarker, and discusses the possible biological role of fucosylation in cancer development.

## 1. Introduction

Glycosylation is involved in a variety of biological phenomena including birth, differentiation, growth, inflammation, carcinogenesis, and cancer metastasis. Each oligosaccharide structure consists of several kinds of monosaccharides, the addition of which is catalyzed by specific glycosyltransferases. Among approximately 10 kinds of oligosaccharide modifications, fucosylation isone of the most important types in cancer. Hakomori *et al*. first reported the involvement of fucosylation in cancer, comparing the fucosylation levels of glycolipids in hepatoma cells and normal hepatocytes [1]. 

Fucose (6-deoxy-L-galactose) is a monosaccharide that is found in glycoproteins and glycolipids present in vertebrates, invertebrates, plants, and bacteria. Fucosylation, which comprises the transfer of a fucose residue to oligosaccharides and proteins, is regulated by many types of molecules, including fucosyltransferases, guanosine diphosphate (GDP)-fucose synthetic enzymes, and GDP-fucose transporter(s). Fucosylation levels in normal liver and colon are relatively low, but increase during carcinogenesis. Most target glycoproteins undergoing fucosylation are secretary proteins or membrane proteins on the cell surface. Increased fucosylated proteins in sera of patients with cancer are dependent on cellular fucosylation of cancer tissues and/or changes in fucosylation states in the liver. 

Glycomics, the systematic study of glycans and glycan-binding proteins in various biological systems, is an emerging field in the post-genomic and post-proteomic era. Change in fucosylation of glycoproteins such as fucosylated alpha-fetoprotein (AFP) is one of the most representative types of glycan-related cancer biomarkers. Increases in fucosylated AFP in sera of patients with hepatocellular carcinoma (HCC) was reported by Breborowicz *et al.* [2,3]. They characterized microheterogeneity of AFP in several liver conditions, and found increases in α1-6 fucosylation (core fucosylation) of AFP using lectin affinity electrophoresis [4,5]. AFP is a well-known tumor marker for HCC, but sometimes also increases in benign liver diseases such as chronic hepatitis and liver cirrhosis. In contrast, AFP with core fucosylation is a very specific marker for HCC [6,7]. AFP with core fucosylation was known as AFP-L3, because it was detected as the L3 fraction on Lensculinaris agglutinin (LCA) lectin electrophoresis. Core fucosylation involving attachment of fucose to the innermost *N*-acetylglucosamine in *N*-glycans is synthesized by α1-6 fucosyltransferase (Fut8) [8]. However, expression of Fut8 is increased in both HCC tissues and surrounding tissues with liver cirrhosis [9]. Therefore, complicated molecular mechanisms might exist in the production of fucosylated glycoproteins in cancer. 

There have been a number of methods to measure AFP-L3. The second generation AFP-L3 assay was based on liquid phase binding of the AFP-L3 glycoform with LCA and two specific monoclonal antibodies [10]. More recently, an automated immunoassay system for AFP-L3 has been developed [11]. This assay was based on an on-chip electrokinetic reaction and separation by affinity electrophoresis. It showed higher sensitivity than conventional methods. Since the limit of detection is 0.1 ng/mL AFP, the assay is available for early HCC detection. Block *et al*. have reported certain types of novel fucosylated glycol-biomarkers for HCC [12]. In a blind test, AFP had a better area under the receiver operating characteristic (ROC) curve than Des-gamma carboxy-prothrombin (DCP) and AFP-L3 in a total of 836 patients with chronic liver diseases and HCC [13]. Although AFP is one of the best standards for detection of HCC, AFP has limited utility for detecting HCC [14], suggesting that a combined assay of AFP, DCP, and AFP-L3 would be recommended as a follow-up for patients with chronic liver diseases. 

Loss of fucosylation has significant biological consequences. Lack of IgG core fucosylation results in 50–100 times higher activity of antibody-dependent cellular cytotoxicity [15]. These data have been confirmed by several groups, and as a result, genetically modified antibodies are now used in clinical trials. Complete loss of fucosylation was found in colon cancer cell line, HCT116 [16]. The loss was due to a GDP-mannose-4,6-dehydratase (GMDS) mutation, resulting in production of undetectable levels of GDP-fucose, a donor substrate for fucosyltransferases. Similar genetic mutations of GMDS were found in various cancer cell lines and human cancer tissues.

However, FX (GDP-4-keto-6-deoxy-mannose-3,5-epimerase-4-reductase) deficient mice showed severe defects in the immune system and died shortly after birth [17]. Both FX and GMDS are rate-limiting enzymes in the *de novo* pathway producing GDP-fucose. Theoretically, FX deficient mice should show more severe abnormalities than Fut8 deficient mice, which lack only core fucose and not total fucose. However, HCT116 cells can grow rapidly under normal conditions, when growth factor receptors in the cells lack fucosylation. Therefore, there may be many genetic mutations which affect the signaling pathway of growth factor receptors in HCT116 cells. For example, autophosporylation of growth factor receptors without ligand stimulation might exist in these cells. 

In this review, we describe novel types of fucosylated cancer biomarkers, possible mechanisms for the production of fucosylated proteins, and biological functions of fucosylation and defucosylation.

## 2. Fucosylated Haptoglobin

**Figure 1:**
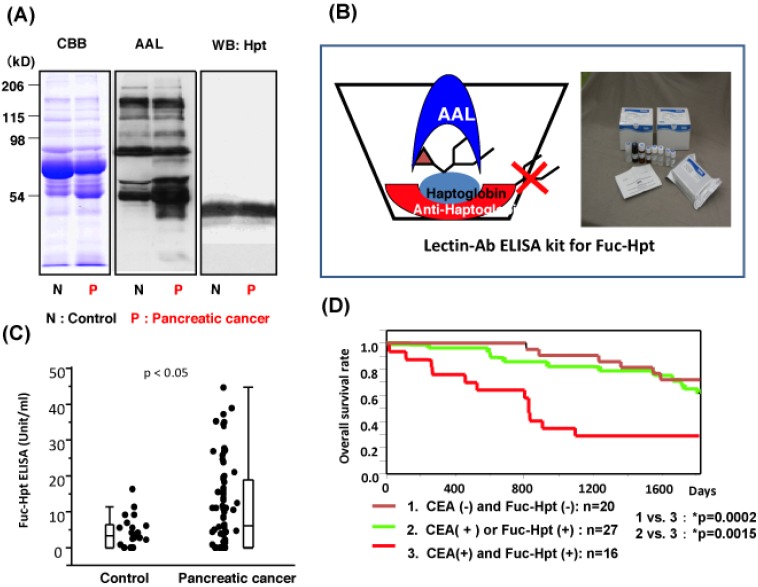
Fucosylated haptoglobin is a novel cancer biomarker for differential diagnosis and predicted prognosis. (**A**) Lectin blot using aleuria aurantia lectin (AAL) detected a protein of approximately 40 kDa from sera of patients with pancreatic cancer. Coomassie Brilliant Blue staining showed no changes in levels of this protein. This figure is cited from reference [22] with slight modification; (**B**) Establishment of lectin-antibody ELISA kit to measure Fuc-Hpt. Schematic system is shown; (**C**) Representative data of the Fuc-Hpt ELISA kit. Seventy-two cases of patients with pancreatic cancer and 22 healthy volunteers were assayed with 25 times dilution of sera. This data is cited from reference [28] with slight modification; (**D**) Combination assay of Fuc-Hpt and carcinoembryonic antigen is a marker for poor prognosis in patients with colorectal cancer after operation. This data is cited from reference [23] with slight modification.

Fucosylated haptoglobin (Fuc-Hpt) was first found in sera of patients with advanced ovarian cancer and breast cancer [18,19]. Ulex europaeus agglutinin (UEA) lectin, which mainly recognizes α1-2 fucose residues, was used to detect Fuc-Hpt. A recent study showed that Fuc-Hpt, present in sera of patients with pancreatic cancer, involved the addition of fucose residues through the α 1-3/1-4 linkage [20]. We found Fuc-Hpt in sera of patients with pancreatic cancer as shown in Figure 1. In addition, fucosylated glycoproteins are recognized by several types of lectins. These lectins include Aleuria aurantia lectin (AAL), UEA, LCA, and Aspergillus oryzae lectin (AOL). AAL recognizes α1-3/α1-4 and α1-6 fucose, UEA recognizes α1-2 fucose, LCA recognizes the native form of α1-6 fucose with a mannose arm, and AOL recognizes a specific form of α1-6 fucose [21]. Recently, a more specific lectin for α1-6 fucose, called Pinellia ternata lectin or PTL, has been isolated from mushrooms (submitted for publication). This lectin could assist in cancer diagnosis. Western blotting of serum samples from patients with pancreatic cancer, using the AAL lectin, showed that a protein of approximately 40 kDa was highly fucosylated. The N-terminal sequence revealed that this protein was the haptoglobin β chain [22]. The fucosylated haptoglobin was found in 60–80% of the patients, and the prevalence increased progressively with stage of the disease. Increased fucosylated haptoglobin levels have been observed in several types of cancer (20–40%). Haptoglobin is produced in the liver and exhibits a low level of fucosylation, since the expression of Fut8 and GDP-fucose synthesis enzymes such as FX and GMDS is quite low in the normal liver. The ectopic expression of haptoglobin is observed in special conditions such as infections, inflammation and cancer. 

Where is fucosylated haptoglobin produced in patients with pancreatic cancer? A pancreatic cancer cell, PSN-1, expresses haptoglobin mRNA and produces fucosylated haptoglobin in conditioned medium. However, this situation is rare in comparison with the prevalence of fucosylated haptoglobin (60–80%). To answer this question, we transplanted a colon cancer cell line, HCT 116 which lacks fucosylation due to GMDS mutation, into athymic mice and investigated serum levels of Fuc-Hpt during tumor development [23]. HCT 116 cells were studied in two different pathways by using intrasplenic and subcutaneous injections. All animals after intrasplenic injection were positive for Fuc-Hpt with macro/micro liver metastasis. In contrast, two of six mice injected subcutaneously were positive for Fuc-Hpt. The positive animals exhibited intraperitoneal invasion of tumor cells. These data suggested that metastatic lesions of cancer could be one of the major sources of Fuc-Hpt production. In the case of HCT116 cells, cancer cells themselves cannot produce Fuc-Hpt, indicating that their surrounding normal cells such as hepatocytes and lymphocytes produced Fuc-Hpt. After we reported that Fuc-Hpt is a promising biomarker for pancreatic cancer, many researchers have reported increases in Fuc-Hpt in sera of patients with different cancers such as colon cancer [24], liver cancer [25], lung cancer [26], and pancreatic cancer [27].

## 3. Development of Lectin-Antibody ELISA for Fuc-Hpt

When evaluating Fuc-Hpt using Western blotting and AAL lectin, two problems occur in examining many samples at the same time. The first is whether fucosylated 40 kDa protein is really haptoglobin and the second is the difficulty in quantitating levels of Fuc-Hpt. To overcome these obstacles, we established a lectin-antibody ELISA to measure Fuc-Hpt. Since most oligosaccharides on IgG are fucosylated, the Fab fragment of IgG is used in this ELISA, after treatment with papain to remove the Fc part of IgG. Detailed procedures have been previously described [28]. When the conditioned medium from PK8 pancreatic cell line, transfected with an expression vector of human haptoglobin, was used for the standard curve of lectin-antibody ELISA, Fuc-Hpt was quantitatively measured in relative units. The cut-off index of Fuc-Hpt was 539 unit/mL, according to the ROC curve, and the sensitivity and the specificity of Fuc-Hpt for diagnosis of pancreatic cancer were 70% and 77%, respectively. In addition, a few cases of pancreatic cancer showed high levels of Fuc-Hpt, even at an early clinical stage. There is a possibility that these cases involved micrometastasis of the liver or lymph node. It is difficult to correlate Fuc-Hpt levels and the prognosis after operation, because the prognosis of pancreatic cancer is quite poor. Therefore, we compared the level of Fuc-Hpt before the operation with the prognosis after the operation in 63 cases of colorectal cancer [23]. An assay using Fuc-Hpt and carcinoembryonic antigen (CEA), a conventional cancer biomarker for colorectal cancer, was a marker for poor prognosis. Both CEA and Fuc-Hpt positive cases showed a poorer prognosis than CEA or Fuc-Hpt positive or both CEA and Fuc-Hpt negative. This data could be directly applied to clinical laboratory examinations. The reason for poor prognosis of colorectal cancer, which produces Fuc-Hpt, could be the micrometastasis as suggested from the results of experiments using HCT116 cells as described above.

## 4. How Are Fucosylated Proteins Produced in Cancer?

As mentioned in the introduction, an increase in cellular fucosylation is dependent on the induction of several glycosyltransferases, GDP-fucose production, and up-regulation of the GDP-fucose transporter. Although expression of these parameters is up-regulated in liver cirrhosis, serum fucosylated proteins are not dramatically increased as in HCC. Fucosylated AFP in HCC is an example. While the GDP-fucose level is up-regulated in HCC tissues compared to surrounding tissues, the level was only 2–3 times higher [29]. Another mechanism might exist to increase fucosylated AFP in serum of HCC patients.

Fucosylated glycoproteins produced in hepatocytes are secreted into the bile, which is on the apical side of hepatocytes [30]. When oligosaccharide structures of bile and serum glycoproteins were compared using lectin blotting or 2D mapping, dramatic increases in fucosylation were observed for bile glycoproteins. In the human liver, Fut6 is involved in the synthesis of Lewis types of fucosylation, and hepatic glycoproteins with this oligosaccharide structure are present in the bile. In mice, Fut6 is a pseudo-gene, and the secretion of certain kinds of hepatic glycoproteins into the bile is disrupted in Fut8 knockout mice. The Fut8 knockout mice show decreased levels of hepatic glycoproteins such as α1-acid glycoprotein and α1-antitrypsin in their bile, suggesting that fucosylation regulates the secretion of certain types of hepatic glycoproteins into the bile. The disruption of this system could be one of the mechanisms underlying the increases in fucosylated protein levels, including AFP-L3 in the serum of patients with HCC. 

It is possible that HCC cells lose their polarity because they are rapidly proliferating. There are often no bile duct structures in HCC tissues. Therefore, selective secretion of fucosylated glycoproteins was not observed in HCC tissues, which led to the production of AFP-L3. AFP-L3 has also been detected in severe acute hepatitis [31]. These hypotheses were confirmed, using a rodent model of hepatocarcinogenesis [32]. Fucosylation of glycoproteins in bile is increased with progression of the liver disease. This could be due to an inflammation. A representative cytokine involved in inflammation, IL6, induces expression of fucosylation regulatory genes [33]. In contrast, serum fucosylation is not significantly increased in chronic liver diseases but increased in HCC. Interestingly, target glycoproteins for fucosylation in HCC are limited in sera and bile, suggesting that fucosylation is a signal for secretion into bile and this selective secretion is dependent on the characteristics of each glycoprotein. The structure as well as the numbers of oligosaccharides attached to proteins might be key factors for selective secretion via glycosylation. These data in part were confirmed with experiments using a human hepatoma cell line, HepG2, which has cellular polarity [34].

## 5. Biological Significance of Fucosylation Loss

**Figure 2:**
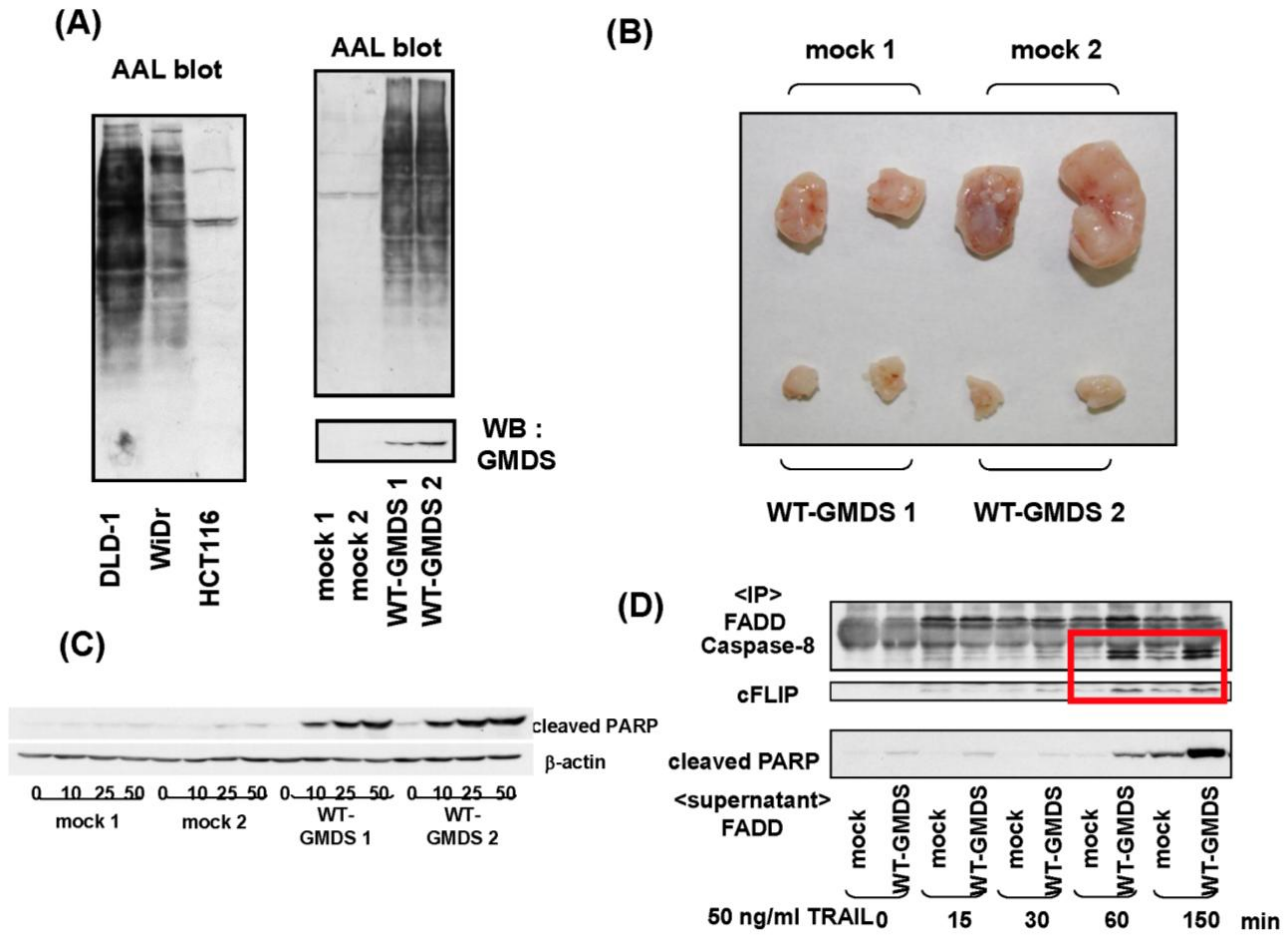
Deficiency of GDP-mannose-4,6-dehydratase (GMDS) leads to escape from natural killer (NK) cell-mediated tumor surveillance through modulation of tumor necrosis factor-related apoptosis-inducing ligand (TRAIL) signaling. (**A**) After transfection of wild-type GMDS gene into HCT116 cells, Western blot analysis of GMDS and AAL was performed. The binding to AAL was restored in transfected cells (WT-GMDS). Each number represents an independent clone; (**B**) Tumor growth of GMDS-rescued cells on the backs of athymic nude mice was significantly suppressed compared to mock cells. Rejected tumors at one month after transplantation were photographed; (**C**) The higher susceptibility of the GMDS-rescued cells to recombinant human TRAIL was confirmed by Western blotting of cleavedpoly (adenosine diphosphate-ribose) polymerase (PARP), which is a signal of apoptosis; (**D**) Complex II formation after TRAIL treatment was inhibited in HCT 116 cells which lacked cellular fucosylation. Immunoprecipitaion of FADD followed by Western blotting of caspase 8, c-Flip, and cleaved PARP were performed on HCT 116 clones treated with TRAIL. Detailed procedures are described in reference [29]. All data were derived from references [11,29].

While many studies have revealed that fucosylation is closely associated with cancer biology through modulation of signal transduction and the cell-cell adhesion pathways, we recently provided new evidence that fucosylation affects tumor immune surveillance via another signaling pathway, tumor necrosis factor-related apoptosis-inducing ligand (TRAIL) signaling [16]. When we examined the global fucosylation level in several colon cancer cells using AAL, which recognizes fucosylated oligosaccharides, little binding to AAL lectin was found in a human colon cancer cell line, HCT116. Further analysis revealed that HCT116 cells had a deleted GMDS transcript, which eliminated their ability to synthesize GDP-fucose, and resulted in an almost complete absence of fucosylation (Figure 2A). Transfection of the wild-type GMDS gene into HCT116 cells restored the cellular fucosylation. GMDS-rescued cells showed dramatically suppressed tumor formation and metastasis compared with mock cells when they were inoculated into athymic nude mice (Figure 2B). Depletion of natural killer (NK) cells stimulated tumor growth of the GMDS-rescued cells, but not that of the mock cells, indicating that a deficiency of fucosylation leads to escape from NK cell-mediated tumor immune surveillance. TRAIL is expressed mainly on the surface of immune cells, where it functions in T-cell homeostasis and NK cell-mediated killing of virally infected or oncogenically transformed cells [35,36]. Binding of TRAIL receptors by the ligand leads to apoptosis through a specific signaling cascade. Subsequent studies revealed that the GMDS-rescued cells were significantly more susceptible to TRAIL-induced apoptosis, which resulted in increased sensitivity of GMDS-rescued cells towards NK cells (Figure 2C). Aberrant transcripts of the GMDS gene were found in three other cancer cell lines as well as several colon and ovarian cancer tissues. Thus, loss of GMDS might be a common mechanism for cancer cells to evade TRAIL-mediated killing. While the increase in fucosylation is important at an early stage of carcinogenesis, defucosylation through genetic mutation in certain types of advanced cancers would lead to escape from NK-cell mediated tumor surveillance, and the acquisition of more malignant characteristics because of their ability to kill cancer cells. Optimized soluble recombinant human TRAIL or agonistic antibodies targeting TRAIL receptors are undergoing phase I or II clinical evaluation as promising proapoptotic antitumor therapeutic agents in patients with several types of tumors [37]. However, it has now become clear that many types of tumor cells are resistant to TRAIL [38,39]. Thus, studies are now underway to identify and characterize potential biomarkers of sensitivity to TRAIL. When several kinds of cancer cell lines are treated with a demethylation agent, zebularine, to induce DNA hypomethylation, expression of fucosylation regulatory genes is up-regulated, resulting in susceptibility to TRAIL-induced apoptosis [40].

Combination therapy of TRAIL and demethylation agents could be a promising immunotherapy. In contrast, we have recently found that loss of fucosylation induced the inhibition of complex II formation which is an important component in apoptosis signaling [41] (Figure 2D). However, complex II, comprised of caspase-8 and cellular Fas-associated death domain [FADD]-like interleukin-1 beta-converting enzyme [FLICE] inhibitory protein, are not glycoproteins. Further studies should clarify how fucosylation affects complex formation. After answering this question, TRAIL therapy could be a more effective means of treatment.

## 6. Conclusions

To use glyco-cancer biomarkers in clinical diagnosis, simple and repeatable methods are required. However, the mechanisms underlying the production of tumor specific oligosaccharide changes should first be investigated. While proteomic/glycomic approaches can produce a variety of candidates for cancer biomarkers, their target glycoproteins for characteristic oligosaccharides should be identified. Although lectins are powerful tools for simple oligosaccharide analyses, their cross-reactivity and low affinity for characteristic oligosaccharides present problems. An antibody for characteristic oligosaccharides or an antibody for characteristic glycol-peptides is a more ideal tool. We have investigated biological functions of cellular fucosylation and its clinical application. Although there are many fucose-specific lectins, their cross reactivity to the linkage of fucose-binding is a problem. Recently, we have found a novel lectin, which can bind to core-fucose, but not other types of fucose (manuscript submitted). This lectin can be used for immunohistochemistry. Since core-fucosylation regulates cell surface glycoproteins such as epidermal growth factor and transforming growth factor beta receptors directly [42,43], and loss of fucosylation brings dramatic changes in TRAIL signaling as described above, fucosylation is a promising target for cancer diagnosis and therapeutics.
